# Explainable and visualizable machine learning model development and validation for 5-year postoperative survival prediction in prostate cancer patients aged ≥ 65 years

**DOI:** 10.1186/s12877-026-07551-2

**Published:** 2026-05-04

**Authors:** Huaying Cheng, Tongping Shen

**Affiliations:** 1 School of Information Engineering, Anhui University of Chinese Medicine, Hefei, China; 2 Department of Urology, The First Affiliated Hospital of University of Science and Technology of China, Hefei, China; 3 Wannan Medical College, Wuhu, China

**Keywords:** Prostate Cancer, Machine learning, Older adults, SHAP, Shiny

## Abstract

**Background:**

Machine Learning (ML) models have achieved outstanding performance in predicting post-surgical survival. However, the “black-box” nature of ML models restricts their clinical application. This study aims to develop and validate a clinically feasible, machine learning-based online prediction system for predicting the 5-year survival status of patients with prostate cancer (PCa) after surgery.

**Methods:**

This study conducted a retrospective analysis of clinical data from 300 older adults with PCa aged ≥ 65 years. LASSO regression, random forest (RF), and recursive feature elimination (RFE) were employed to screen for clinical parameters. Subsequently, the cohort was split into a training set and a test set at a 7:3 ratio, and 25 machine learning approaches were utilized for comparative assessment to determine the optimal model. Calibration curves were applied to evaluate the performance of each model, while decision curve analysis (DCA) was adopted to assess their clinical usefulness. In addition, SHAP (Shapley Additive exPlanations) values were used to interpret the model features. Finally, the Shiny framework was employed to develop an online prediction system.

**Results:**

The intersection of the three feature selection algorithms identified 13 clinical parameters: Age, ALB, BUN, CRE, HB, PLT, PT, PSA, GS, PNI, PSM, pT and pN. Comparing 25 machine learning algorithms, LightGBM gave the best results. Its performance metrics were: Accuracy 0.9328, Sensitivity 0.9074, Specificity 0.9538, Positive Predictive Value (PPV) 0.9418, Negative Predictive Value (NPV) 0.9118, AUC 0.9778, Recall 0.9074, F1 Score 0.9143. SHAP values revealed the contribution of each feature in the LightGBM model.

**Conclusion:**

This study successfully developed a 5-year postoperative survival prediction model for prostate cancer patients. The model demonstrated favorable predictive performance in the test set, which may provide a reference for clinical decision-making. Further multi-center external validation is required to clarify its clinical application value in the future.

**Supplementary Information:**

The online version contains supplementary material available at 10.1186/s12877-026-07551-2.

## Introduction

PCa is the most common malignant tumor of the urinary system and the second most common malignant tumor in men, ranking fifth in terms of mortality rate [1–3].

GBD 2023 data show that the global death toll from prostate cancer was 21,461,648 in 1990 and 47,295,326 in 2023, indicating that it has become an important public health issue [4].

PCa is a complex disease that poses significant challenges to both patients and clinicians. High heterogeneity is one of its core characteristics. Within the same patient’s prostate tissue, different lesions may exhibit distinct biological behaviors (e.g., aggressive vs. indolent). Among different patients, the mutation profiles, epigenetic modifications, and tumor molecular subtypes also show significant differences [5]. This heterogeneity leads to significant uncertainty in the natural course of PCa. While the majority of PCa presents as slowly progressive indolent tumors, a minority of patients may develop into highly malignant diseases characterized by strong invasiveness and distant metastasis.

Treatment decisions are primarily based on a comprehensive assessment of the patient’s prognostic risk, including clinical-pathological features and individual patient health conditions. Clinical pathological features include prostate-specific antigen (PSA) levels, tumor Gleason scores, and TNM staging. Individual patient health conditions encompass sociodemographic and clinical characteristics such as age, overall health status (e.g., presence of other chronic diseases), and life expectancy [6, 7].

Additionally, in recent years, notable advances have been achieved in the diagnosis and treatment of PCa, bringing substantial innovations to clinical practice. Considering the marked variations in disease progression stages, individual physiological conditions, and quality-of-life expectations among PCa patients, both clinical research and practical experience have verified that surgical treatment can effectively prevent or delay tumor recurrence and progression, thereby significantly enhancing patients’ disease-free survival and overall survival rates.

In addition to surgical treatment, clinical practice offers a variety of non-surgical options, including external beam radiotherapy, brachytherapy, chemotherapy, hormone therapy, and targeted drug therapy, providing patients with a diverse range of treatment choices [8].

It is crucial to formulate an individualized optimal treatment plan for each patient. This decision directly impacts disease control efficacy and patient prognosis, while also helping to optimize medical resource allocation and alleviate patients’ daily and financial care burdens [9].

Prostate cancer has an extremely high incidence rate among older adults. The incidence and mortality rates of this disease are strongly age-related, with the average age at diagnosis for most patients centered around 65 years old. The formation of this age-related difference may be attributed to the joint effects of multiple factors, including genetic susceptibility, environmental exposure, and socioeconomic factors [10].

In 2015, 72,000 new prostate cancer cases and roughly 30,700 deaths were documented in China. While the incidence of prostate cancer in China is substantially lower than that in European and American countries, it has exhibited an upward tendency in recent years, with a faster growth rate compared to developed nations in Europe and America [11]. By 2022, the number of new cases and deaths from prostate cancer in China is projected to increase to 134,200 and 47,500 cases, respectively [12].

China still confronts numerous challenges in the early diagnosis and standardized treatment of prostate cancer, exhibiting a notable disparity compared with Western developed countries. Taking the United States as an illustration, nearly 91% of newly diagnosed prostate cancer cases in the country are clinically localized, with radical prostatectomy or radical radiotherapy serving as the first-line treatment. Patients receiving standardized therapy tend to have a favorable prognosis, with a 5-year overall survival rate approaching 100%. In contrast, only 30% of newly diagnosed cases in China are clinically localized prostate cancer, while the remaining 70% are patients with locally advanced or metastatic disease. These patients are ineligible for local curative treatment, resulting in a relatively poor prognosis [13].

At present, treatment guidelines for PCa primarily rely on traditional linear models—such as survival analysis and Cox proportional hazards models—for risk stratification. Nevertheless, owing to the intricate nonlinear interactions among various prognostic factors in PCa biology, relying exclusively on these linear approaches may not be adequate to precisely forecast individual survival outcomes.

As artificial intelligence (AI) and ML technologies continue to advance, enabling the processing of massive datasets in relatively short periods, their applications in the medical field are increasingly widespread. Machine learning can be applied to various areas, including drug development, gene expression profiling, biomarker research in multi-omics construction and analysis, and digital pathology slide analysis [14, 15]. In the era of personalized medicine, researchers are developing machine learning algorithms based on individual data to achieve precise predictions of survival outcomes for prostate cancer patients.

Machine learning technology has enormous potential for designing personalized treatment plans [16–18]. By systematically integrating individual factors such as age, health status, and lifestyle, machine learning models can help researchers capture subtle differences in tumor characteristics among prostate cancer patients, thereby providing robust technical support for the formulation of precise, personalized treatment strategies.

Nevertheless, the field still faces considerable challenges. First, the variables adopted in previous studies are insufficient in dimensionality and coverage [19], which reduces model predictive performance and further compromises the reliability of research findings and the accuracy of survival prediction [20]. Second, the small sample sizes and concentrated data sources from single research centers have led to generally diminished representativeness and generalizability of previous research. Finally, the machine learning models currently employed often depend on a single or limited set of model types, with a dearth of comprehensive comparisons across multiple models. This may restrict their generalizability and adaptability in practical, complex clinical scenarios [21].

This study seeks to establish a more accurate and reliable prostate cancer treatment recommendation model using patient data from the First Affiliated Hospital of the University of Science and Technology of China. It integrates machine learning algorithms with SHAP value interpretation techniques, with a specific focus on optimizing survival outcomes for Chinese patients. SHAP technology clarifies the complex associations between features and predictions, enhances model interpretability, and provides valuable references for clinical decision-making. This, in turn, facilitates the optimization of treatment efficacy and ultimately improves the quality of life and long-term survival of Chinese prostate cancer patients.

## Materials and methods

### Data sources and study population

This study consecutively recruited patients who underwent radical prostatectomy (RP) from the First Affiliated Hospital of the University of Science and Technology of China between January 2013 and June 2020. The postoperative survival outcomes of the patients were obtained through a single standardized telephone follow-up conducted on July 1, 2025. The pre-specified endpoint adjudication criteria were as follows: patients were adjudicated as “5-year postoperative mortality” if they died before the individual-specific date of completing 5 years after surgery; patients who were alive on the individual-specific date of 5 years post-surgery, or died after this date, were adjudicated as “5-year postoperative survival”.

We performed strict case screening and data cleaning prior to formal analysis. A total of 188 patients were excluded: 86 cases with incomplete clinical records or missing key variables that precluded valid analysis, and 102 cases who did not meet the age inclusion criterion (younger than 65 years). This resulted in a final analytical cohort of 300 patients with complete follow-up data. The study was approved by the institutional review board (IRB), and all subjects provided written informed consent.

### Data preprocessing and feature selection

In the original data of this study, there was a marked imbalance in sample size between Group 0 (*n* = 234) and Group 1 (*n* = 66). This imbalance may cause the model to develop a prediction bias towards the majority class, and reduce the model’s ability to identify the minority class. To address this issue, we adopted the Synthetic Minority Oversampling Technique (SMOTE) to perform class balancing on the original dataset. SMOTE was implemented using the DMwR package (version 0.4.1) in R, with the core parameter set as k = 5 (the 5-nearest neighbor algorithm was applied to generate synthetic samples), to ensure that the feature distribution of the synthetic samples was consistent with that of the original minority class.

**Fig. 1:**
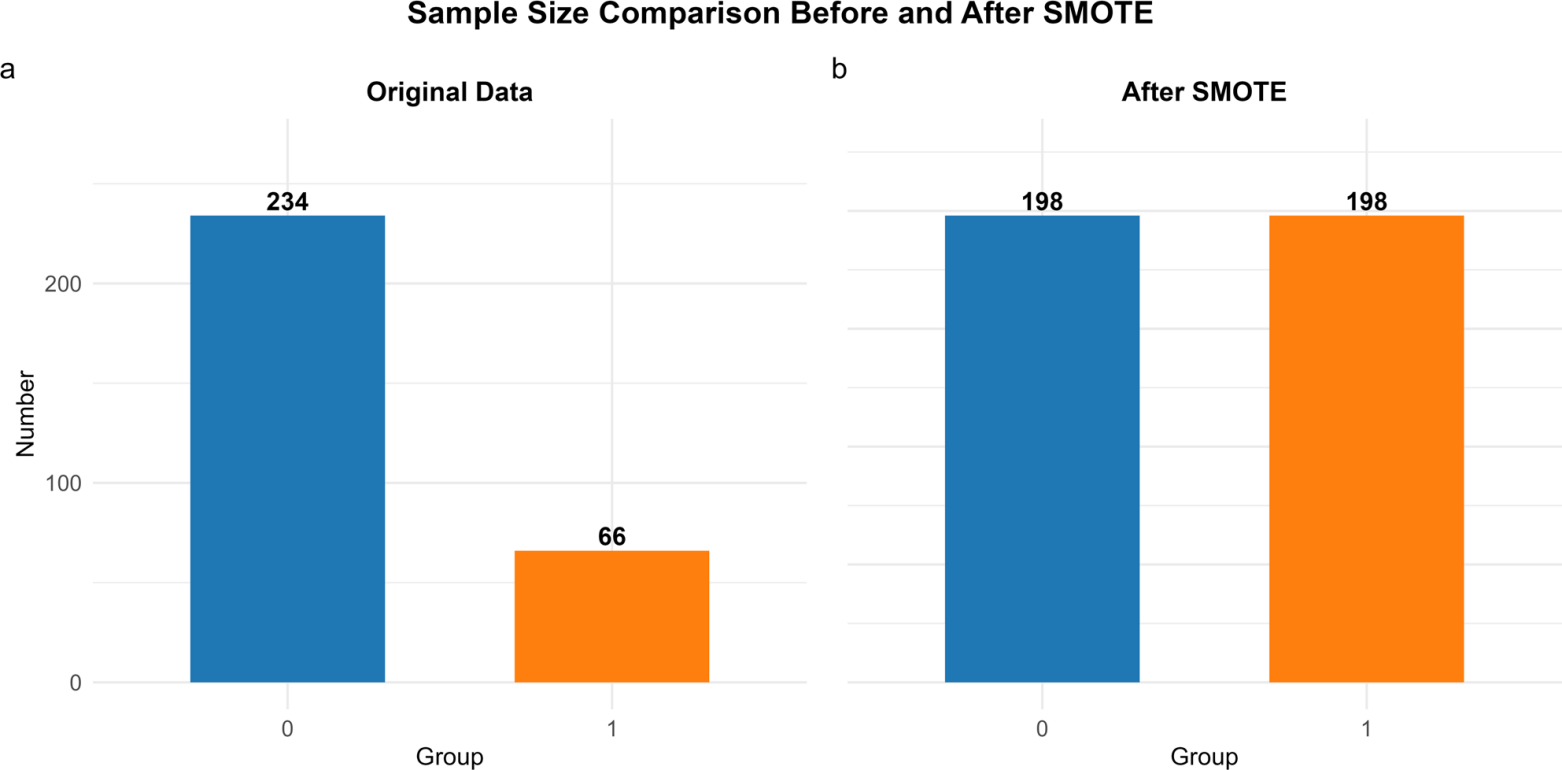
Sample Size Comparison Before and After PSM. (a) Original Data; (b) After SMOTE

After SMOTE processing, the sample sizes of both groups were balanced and adjusted to 198 cases (as shown in Figs. 1(a) and 1(b)), successfully achieving balanced matching between groups and laying a more reasonable dataset foundation for subsequent model analysis.

**Fig. 2:**
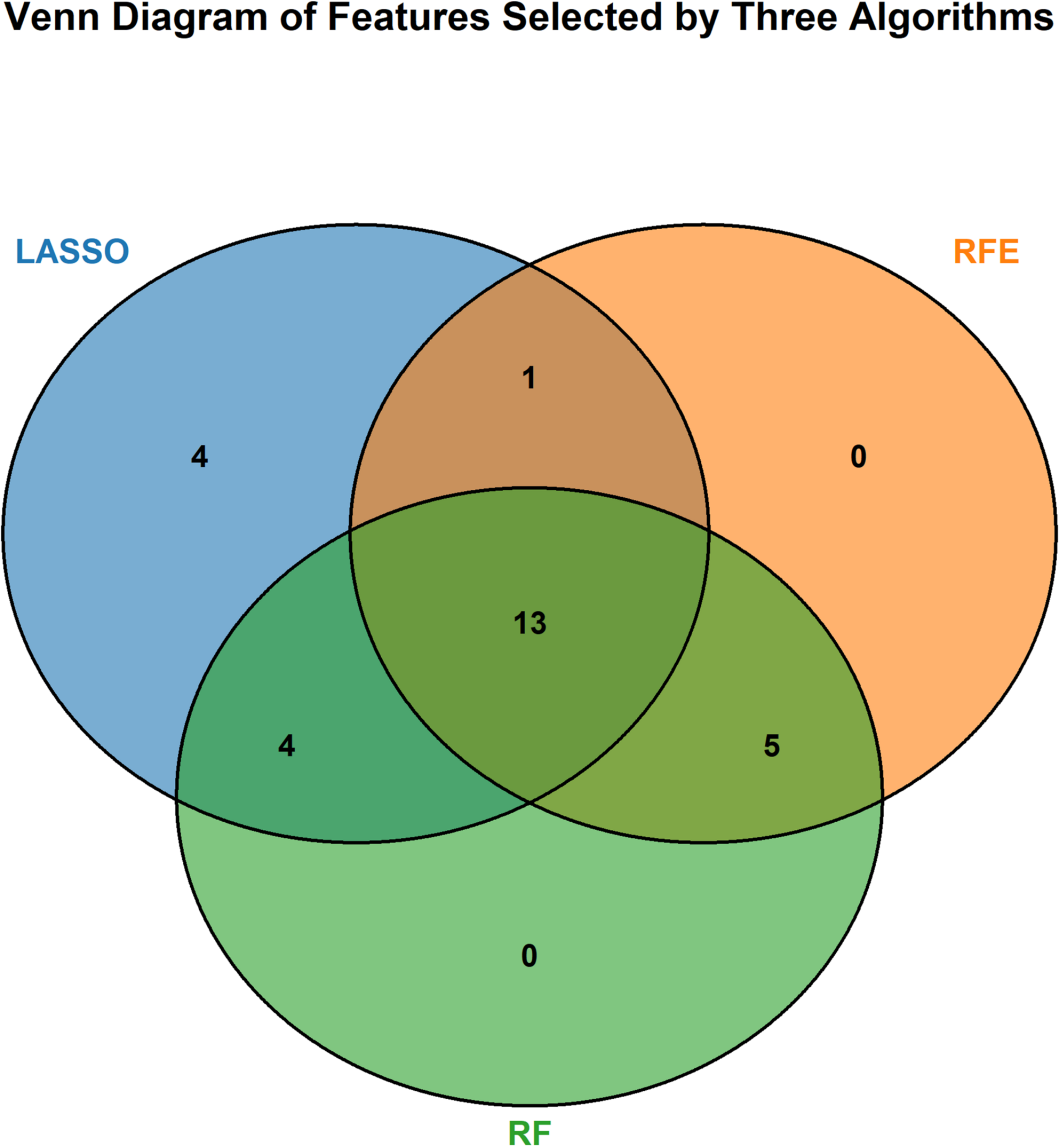
Venn diagram of features selected by three algorithms

We employed three feature selection algorithms, namely LASSO regression, RF, and RFE. To enhance the reliability of the selected features, we used cross-validation across multiple algorithms. The Venn diagram distribution of the feature selection results is shown in Fig. 2.

LASSO incorporates an L1 regularization penalty term built upon classical linear regression, which forces the coefficients of less relevant features to zero, thereby achieving feature selection and dimensionality reduction. Its advantage lies in the efficient elimination of redundant features. There were 4 features uniquely identified by LASSO alone. In addition, LASSO also participated in multi-algorithm overlapping feature selection, with 4 features jointly identified with RF, 1 feature jointly identified with RFE, and 13 core features jointly identified by all three algorithms.

RF is an ensemble-based feature selection method that selects high-contribution features by calculating feature importance metrics (e.g., the Gini coefficient). Its advantages include the ability to capture nonlinear relationships between features and strong resistance to overfitting. RF serves as an intermediate algorithm connecting LASSO and RFE. It not only participated in the overlapping feature selection of multiple algorithms but also, together with the other two algorithms, determined the core feature set.

RFE iteratively removes the “least important features” and evaluates the performance of feature subsets using cross-validation to determine the optimal number of features. Its advantage is high automation, which can reduce the subjectivity of feature selection. The number of features uniquely identified by RFE alone was 0. All features selected by RFE were included in the multi-algorithm overlapping segments, with 1 feature jointly identified with LASSO, 5 features jointly identified with RF, and 13 core features jointly identified by all three algorithms.

### Model development and evaluation

In this study, we selected 25 machine learning algorithms, covering linear models, tree-based models, ensemble models, and neural network models, including Random Forest, Support Vector Machine, Bayesian GLM, Naive Bayes, K-Nearest Neighbors, Neural Network, Flexible Discriminant Analysis, Gradient Boosting Machine, Classification and Regression Tree, Elastic Net, Logistic Regression, LASSO Regression, Ridge Regression, Linear Discriminant Analysis, Quadratic Discriminant Analysis, C50 Decision Tree, Conditional Inference Tree, Partial Least Squares DA, Bagging Decision Trees, XGBoost, Gaussian Process, Sparse Linear Discriminant Analysis, AdaBoost, Deep Neural Network, and LightGBM.

Our objective is to address the limitations of oversimplified models in current research by comprehensively exploring data features and capturing complex relationships. All analyses in this paper were primarily performed using R software (version 4.3.0). Packages such as plotROC, caret, pROC, and e1071 were utilized for tasks including plotting ROC curves and training machine learning models. Additionally, SHAP plots were generated to visualize clinical features and display each feature’s contribution to the model. Finally, an online prediction system was developed using the Shiny framework to assess patients’ 5-year postoperative survival.

The dataset was randomly split into a training set and a test set, with 70% allocated to the training set and 30% to the test set. Each model was subjected to 10-fold cross-validation and parameter optimization. The evaluation metrics comprised: AUC (Area Under the Curve), AUC_SD (AUC Standard Deviation), Accuracy, Kappa, Sensitivity, Specificity, PPV, NPV, Precision, Recall, and F1.

### Model interpretation and clinical applications

We employed a SHAP-based interpretation approach to analyze and explore the model with the optimal overall performance, aiming to elucidate its predictive mechanism and feature contributions. Derived from cooperative game theory, SHAP theory offers a rigorous and highly interpretable tool to quantify the specific impact and relative importance of each feature on the model’s predictions.

Shiny is a lightweight web framework within the R language ecosystem. It allows rapid encapsulation of R-based models and visualization results into interactive web applications without specialized knowledge, enabling non-technical personnel to access them directly in a web browser. We have developed a “Prostate Cancer Patient 5-Year Postoperative Survival Prediction System” based on Shiny. Its core functionality enables users to input clinical indicators (such as age, PSA, etc.) and obtain, with a single click, predictions of 5-year postoperative survival outcomes and visualizations of feature impacts. This system assists physicians in making clinical decisions.

### Statistical analysis

**Fig. 3:**
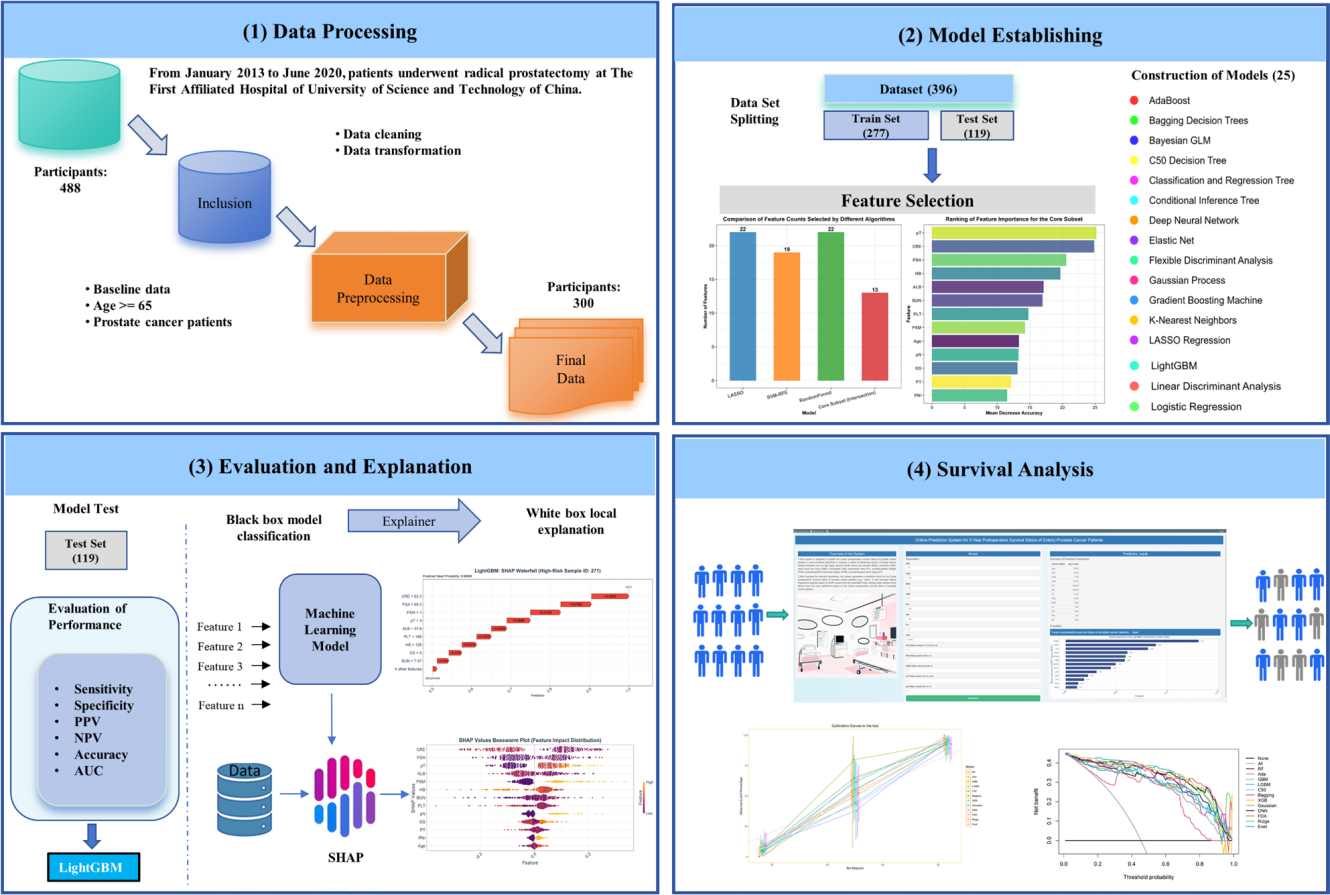
Framework of the prostate cancer survival prediction model

Rigorous statistical methods were employed for data analysis in this study. Continuous variables were presented as mean ± standard deviation or median (interquartile range) based on their distribution characteristics, with intergroup comparisons performed using Student’s t-test or Wilcoxon rank-sum test; categorical variables were expressed as case numbers and percentages, and intergroup differences were evaluated via chi-square test or Fisher’s exact test. Model performance was assessed using discriminative metrics, including sensitivity, specificity, accuracy, and area under the curve (AUC), and 95% confidence intervals for these metrics were constructed using the DeLong method. For survival analysis, the Cox proportional hazards model was adopted, and time-dependent AUC was calculated. The statistical significance threshold was set at *P* < .05. Fig. 3 depicts the establishment process of the prostate cancer survival prediction model and the principles of survival analysis.

## Results

### Baseline characteristics

This study included 300 patients with prostate cancer (PCa) for a comprehensive retrospective analysis. According to the 5-year postoperative survival status, the cohort was evenly divided into the survival group and the death group, with 198 cases in each group (50% each). Table 1 summarizes the baseline clinical characteristics of the included PCa patients.

**Table 1:** Prostate cancer patients’ characteristics stratified by survival status

Characteristic	Statistic	alive (*N* = 198)	dead (*N* = 198)	OR (univariable)	OR (multivariable)
Age	Mean ± SD	71.5 ± 4.5	73.0 ± 4.4	1.08 (1.03–1.13, *p*=.001)	1.17 (1.06–1.30, *p*=.001)
BMI	Mean ± SD	23.5 ± 2.8	22.3 ± 2.7	0.86 (0.80–0.93, *p*<.001)	0.77 (0.66–0.91, *p*=.002)
TBIL	Mean ± SD	14.0 ± 4.9	13.3 ± 3.8	0.97 (0.92–1.01, *p*=.134)	0.96 (0.86–1.07, *p*=.493)
DBIL	Mean ± SD	4.7 ± 2.0	4.5 ± 1.6	0.94 (0.84–1.05, *p*=.271)	
IBIL	Mean ± SD	9.4 ± 3.7	9.0 ± 2.8	0.96 (0.91–1.02, *p*=.228)	
ALB	Mean ± SD	40.7 ± 5.4	37.5 ± 3.6	0.84 (0.79–0.89, *p*<.001)	0.77 (0.67–0.88, *p*<.001)
BUN	Mean ± SD	6.2 ± 1.5	7.1 ± 2.0	1.45 (1.26–1.68, *p*<.001)	2.02 (1.49–2.75, *p*<.001)
CRE	Mean ± SD	75.7 ± 14.0	66.6 ± 14.8	0.95 (0.94–0.97, *p*<.001)	0.92 (0.89–0.95, *p*<.001)
UA	Mean ± SD	349.3 ± 84.3	311.0 ± 87.4	0.99 (0.99-1.00, *p*<.001)	1.00 (0.99-1.00, *p*=.626)
A_G	Mean ± SD	1.5 ± 0.2	1.5 ± 0.2	0.39 (0.15–1.04, *p*=.059)	1.32 (0.12–14.08, *p*=.819)
WBC	Mean ± SD	6.0 ± 1.6	6.1 ± 1.4	1.04 (0.91–1.19, *p*=.551)	
NE	Mean ± SD	3.6 ± 1.3	3.7 ± 1.4	1.06 (0.91–1.22, *p*=.464)	
LYM	Mean ± SD	3.0 ± 12.5	1.7 ± 0.5	0.78 (0.55–1.12, *p*=.177)	1.46 (0.40–5.36, *p*=.565)
NOM	Mean ± SD	0.5 ± 0.2	0.5 ± 0.1	6.48 (1.80-23.32, *p*=.004)	1.43 (0.01-318.98, *p*=.896)
LMR	Mean ± SD	6.5 ± 22.6	3.6 ± 1.6	0.81 (0.71–0.92, *p*<.001)	0.81 (0.40–1.64, *p*=.553)
NLR	Mean ± SD	2.3 ± 1.8	2.5 ± 1.5	1.07 (0.94–1.21, *p*=.301)	
PLR	Mean ± SD	114.4 ± 62.9	122.7 ± 48.7	1.00 (1.00-1.01, *p*=.150)	1.00 (1.00-1.01, *p*=.331)
RBC	Mean ± SD	4.4 ± 0.5	4.1 ± 0.4	0.24 (0.14–0.40, *p*<.001)	1.86 (0.26–13.16, *p*=.535)
HB	Mean ± SD	134.8 ± 13.4	125.9 ± 12.0	0.94 (0.93–0.96, *p*<.001)	0.97 (0.90–1.04, *p*=.346)
PLT	Mean ± SD	184.3 ± 59.7	187.7 ± 39.8	1.00 (1.00-1.01, *p*=.507)	
PT	Mean ± SD	11.5 ± 1.9	11.3 ± 1.3	0.96 (0.85–1.08, *p*=.488)	
FIB	Mean ± SD	2.9 ± 0.7	2.9 ± 0.7	0.92 (0.68–1.23, *p*=.565)	
PSA	Mean ± SD	33.2 ± 28.1	57.0 ± 69.2	1.02 (1.01–1.02, *p*<.001)	1.01 (0.99–1.02, *p*=.390)
GS	1	43 (21.7%)	15 (7.6%)		
	2	37 (18.7%)	37 (18.7%)	2.87 (1.36–6.03, *p*=.005)	0.65 (0.17–2.50, *p*=.526)
	3	72 (36.4%)	26 (13.1%)	1.04 (0.49–2.17, *p*=.927)	0.21 (0.05–0.85, *p*=.029)
	4	19 (9.6%)	45 (22.7%)	6.79 (3.06–15.04, *p*<.001)	0.72 (0.16–3.19, *p*=.667)
	5	26 (13.1%)	70 (35.4%)	7.72 (3.68–16.18, *p*<.001)	1.95 (0.47–8.13, *p*=.358)
	6	1 (0.5%)	5 (2.5%)	14.33 (1.55-132.77, *p*=.019)	4.24 (0.10-173.02, *p*=.446)
PNI	0	157 (79.3%)	85 (42.9%)		
	1	41 (20.7%)	113 (57.1%)	5.09 (3.26–7.94, *p*<.001)	1.99 (0.85–4.68, *p*=.114)
PSM	0	178 (89.9%)	116 (58.6%)		
	1	20 (10.1%)	82 (41.4%)	6.29 (3.66–10.82, *p*<.001)	6.56 (2.28–18.84, *p*<.001)
SVI	0	175 (88.4%)	112 (56.6%)		
	1	23 (11.6%)	86 (43.4%)	5.84 (3.48–9.80, *p*<.001)	3.61 (1.31-10.00, *p*=.013)
pT	2	128 (64.6%)	39 (19.7%)		
	3	68 (34.3%)	107 (54%)	5.16 (3.23–8.26, *p*<.001)	3.93 (1.56–9.92, *p*=.004)
	4	2 (1%)	52 (26.3%)	85.33 (19.89-366.16, *p*<.001)	26.82 (4.29-167.86, *p*<.001)
pN	0	194 (98%)	143 (72.2%)		
	1	4 (2%)	55 (27.8%)	18.65 (6.61–52.66, *p*<.001)	9.57 (1.77–51.67, *p*=.009)
Hypertension	0	108 (54.5%)	116 (58.6%)		
	1	90 (45.5%)	82 (41.4%)	0.85 (0.57–1.26, *p*=.417)	
Diabetes_mellitus	0	180 (90.9%)	156 (78.8%)		
	1	18 (9.1%)	42 (21.2%)	2.69 (1.49–4.87, *p*=.001)	6.77 (1.98–23.18, *p*=.002)

Univariate and multivariate logistic regression analyses were performed to identify factors associated with 5-year mortality. The results demonstrated that elevated age (multivariate OR = 1.17, 95% CI: 1.06–1.30, *p* = .001), increased blood urea nitrogen (BUN) (multivariate OR = 2.02, 95% CI: 1.49–2.75, *p* < .001), prostate-specific membrane antigen positivity (PSM = 1) (multivariate OR = 6.56, 95% CI: 2.28–18.84, *p* < .001), seminal vesicle invasion (SVI = 1) (multivariate OR = 3.61, 95% CI: 1.31–10.00, *p* = .013), advanced tumor stage (pT3/pT4) (pT3: multivariate OR = 3.93, 95% CI: 1.56–9.92, *p* = .004; pT4: multivariate OR = 26.82, 95% CI: 4.29–167.86, *p* < .001) and lymph node metastasis (pN = 1) (multivariate OR = 9.57, 95% CI: 1.77–51.67, *p* = .009) were independent risk factors for 5-year mortality in PCa patients.

In contrast, higher body mass index (BMI) (multivariate OR = 0.77, 95% CI: 0.66–0.91, *p* = .002), elevated albumin (ALB) (multivariate OR = 0.77, 95% CI: 0.67–0.88, *p* < .001) and increased serum creatinine (CRE) (multivariate OR = 0.92, 95% CI: 0.89–0.95, *p* < .001) were confirmed as independent protective factors for 5-year survival (all *p* < .05).

Notably, nutritional risk index positivity (PNI = 1) and hemoglobin (HB) were associated with survival outcomes in univariate analysis but failed to exhibit independent associations after adjusting for confounding factors (multivariate *p* = .114 and 0.346, respectively). Total bilirubin (TBIL) showed no significant correlation with survival status in either univariate or multivariate analysis (*p* > .05). Although uric acid (UA) and certain subgroups of Gleason score (GS) were correlated with survival in univariate analysis, their independent predictive value was not verified in the multivariate model.

Regarding comorbidities, hypertension was not significantly associated with 5-year survival status in PCa patients (*p* = .417). In contrast, diabetes mellitus was identified as an independent risk factor for 5-year mortality (multivariate OR = 6.77, 95% CI: 1.98–23.18, *p* = .002), rather than showing no statistical relevance.

## Feature extraction

**Fig. 4:**
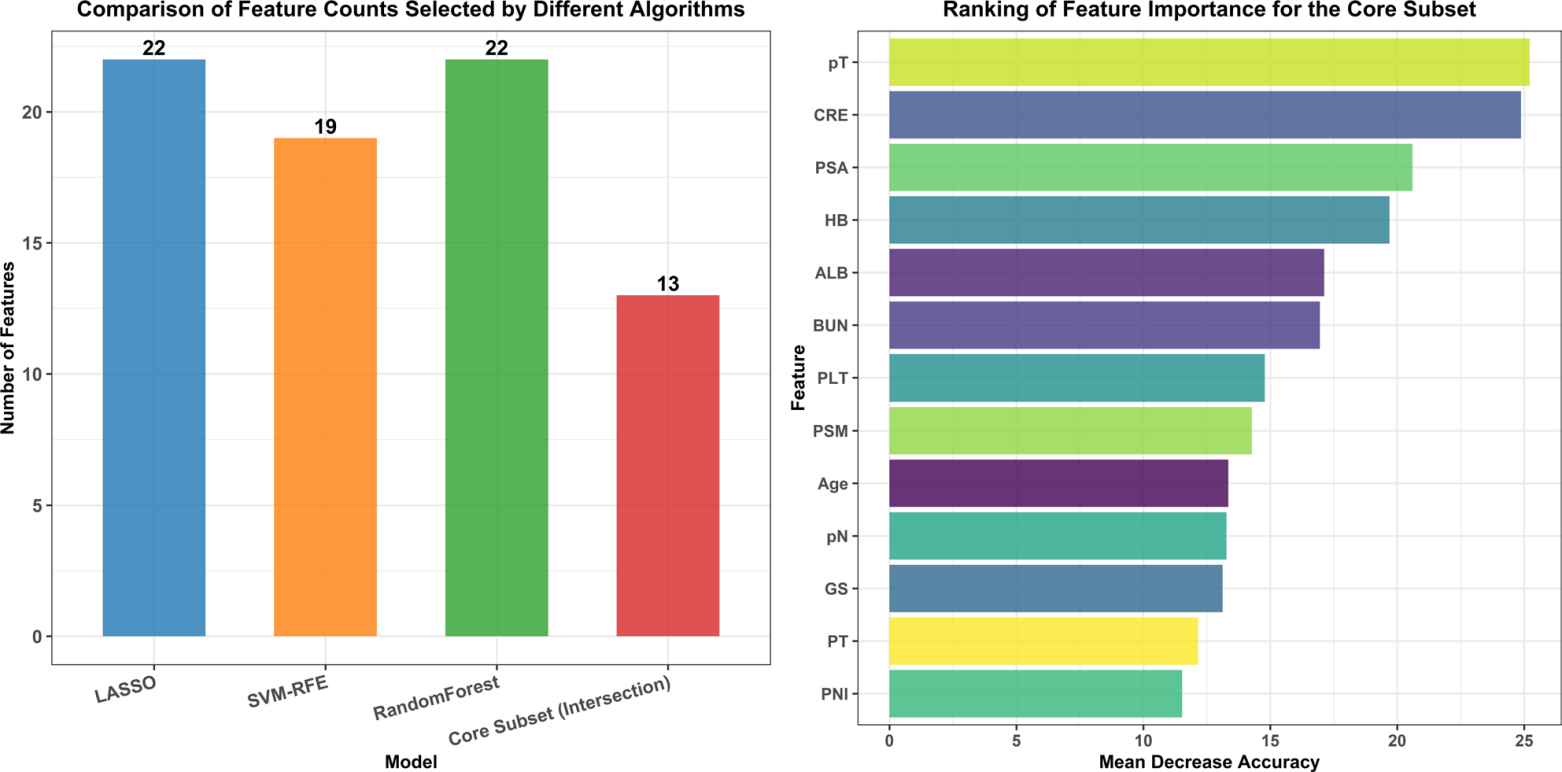
Optimal feature selection process for the merging of three algorithms

Fig. 4 depicts the optimal feature selection process combining LASSO regression, RF, and RFE. The feature selection process for each individual algorithm is presented in Supplementary Fig. 1: Feature Selection Process of the Three Algorithms. We ultimately selected 13 features common to all three models (Age, ALB, BUN, CRE, HB, PLT, PT, PSA, GS, PNI, PSM, pT and pN) as input parameters for machine learning.

### Model construction and selection

**Fig. 5:**
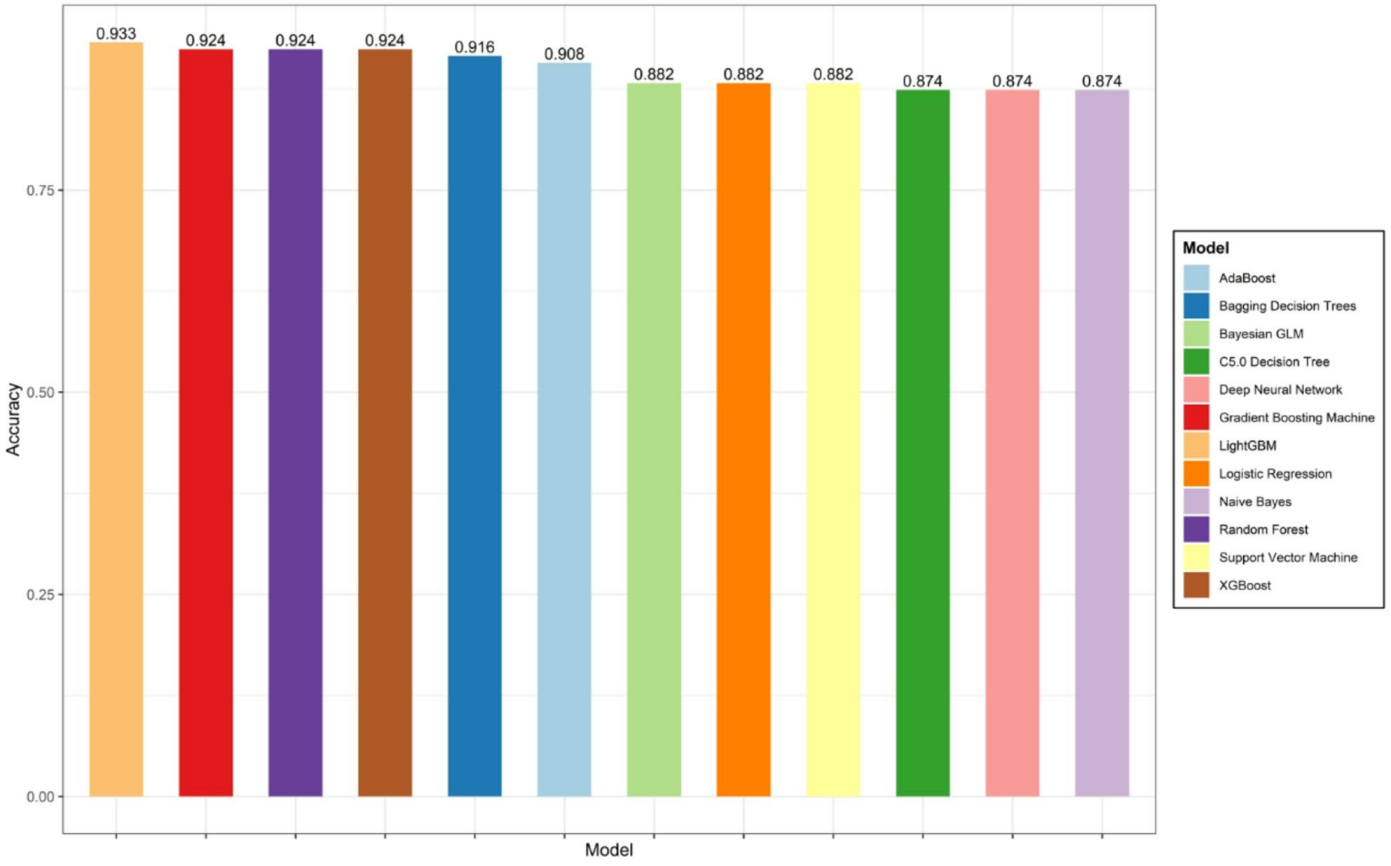
Accuracy of 12 machine learning models

**Fig. 6:**
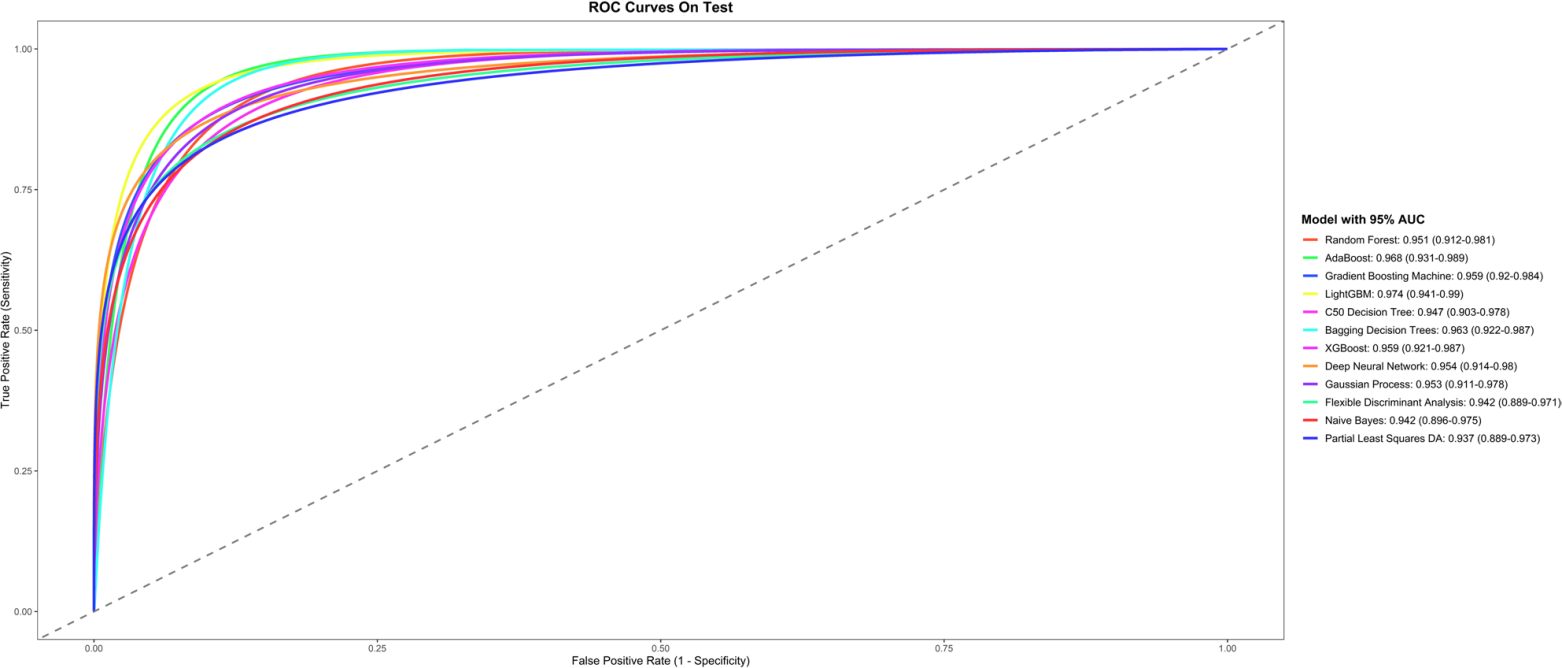
ROC Curves on test for 12 machine learning models

The model results showed that the LightGBM model achieved the highest Accuracy, with a comprehensive optimal performance metric of 0.9328 ( 0.8824, 0.9748), as illustrated in Fig. 5. On the test set, LightGBM, AdaBoost, and XGBoost demonstrated stronger generalization ability, with their area under the receiver operating characteristic curve (AUC) values being 0.9778 (0.9577, 0.9979), 0.9758 (0.9548, 0.9968), and 0.9658 (0.9348, 0.9968), respectively, as shown in Fig. 6 andTable 2. We also compared and validated the performance of all models on both the training set and test set, with detailed performance metrics presented in the first table of Supplementary Table 1.

**Table 2:** Comprehensive performance metrics for 25 machine learning models

Model	AUC_SD	AUC	Accuracy	Kappa	Sensitivity	Specificity	PPV	NPV	Precision	Recall	F1
Gradient Boosting Machine	0.016	0.9632 (0.9340, 0.9925)	0.9244 (0.8739, 0.9664)	0.8640 (0.7645, 0.9478)	0.9074 (0.8235, 0.9796)	0.9538 (0.8983, 1.0000)	0.9423 (0.8723, 1.0000)	0.9254 (0.8596, 0.9846)	0.9423 (0.8723, 1.0000)	0.9074 (0.8235, 0.9796)	0.9245 (0.8660, 0.9697)
LightGBM	0.0189	0.9778 (0.9577, 0.9979)	0.9328 (0.8824, 0.9748)	0.8467 (0.7373, 0.9324)	0.8889 (0.7885, 0.9623)	0.9538 (0.8983, 1.0000)	0.9412 (0.8679, 1.0000)	0.9118 (0.8356, 0.9706)	0.9412 (0.8679, 1.0000)	0.8889 (0.7885, 0.9623)	0.9143 (0.8511, 0.9630)
XGBoost	0.0148	0.9658 (0.9348, 0.9968)	0.9244 (0.8739, 0.9664)	0.8477 (0.7468, 0.9320)	0.9259 (0.8491, 0.9821)	0.9231 (0.8507, 0.9734)	0.9091 (0.8276, 0.9688)	0.9375 (0.8730, 0.9857)	0.9091 (0.8276, 0.9688)	0.9259 (0.8491, 0.9821)	0.9174 (0.8598, 0.9623)
Random Forest	0.0159	0.9547 (0.9191, 0.9903)	0.9244 (0.8739, 0.9664)	0.8467 (0.7433, 0.9326)	0.8889 (0.8000, 0.9643)	0.9538 (0.8983, 1.0000)	0.9412 (0.8695, 1.0000)	0.9118 (0.8382, 0.9714)	0.9412 (0.8695, 1.0000)	0.8889 (0.8000, 0.9643)	0.9143 (0.8515, 0.9643)
Bagging Decision Trees	0.0341	0.9641 (0.9331, 0.9951)	0.9160 (0.8655, 0.9580)	0.8305 (0.7190, 0.9159)	0.9074 (0.8182, 0.9744)	0.9231 (0.8529, 0.9841)	0.9074 (0.8235, 0.9792)	0.9231 (0.8513, 0.9788)	0.9074 (0.8235, 0.9792)	0.9074 (0.8182, 0.9744)	0.9074 (0.8431, 0.9573)
AdaBoost	0.0113	0.9758 (0.9548, 0.9968)	0.9076 (0.8487, 0.9580)	0.8127 (0.6956, 0.9145)	0.8704 (0.7708, 0.9535)	0.9385 (0.8679, 0.9857)	0.9216 (0.8421, 0.9818)	0.8971 (0.8225, 0.9661)	0.9216 (0.8421, 0.9818)	0.8704 (0.7708, 0.9535)	0.8952 (0.8222, 0.9515)
Support Vector Machine	0.0608	0.9296 (0.8817, 0.9776)	0.8824 (0.8235, 0.9328)	0.7619 (0.6301, 0.8648)	0.8519 (0.7419, 0.9423)	0.9077 (0.8281, 0.9706)	0.8846 (0.7800, 0.9623)	0.8806 (0.7931, 0.9516)	0.8846 (0.7800, 0.9623)	0.8519 (0.7419, 0.9423)	0.8679 (0.7865, 0.9273)
Bayesian GLM	0.0675	0.9282 (0.8781, 0.9783)	0.8824 (0.8233, 0.9328)	0.7612 (0.6264, 0.8655)	0.8333 (0.7199, 0.9286)	0.9231 (0.8485, 0.9844)	0.9000 (0.8043, 0.9778)	0.8696 (0.7794, 0.9467)	0.9000 (0.8043, 0.9778)	0.8333 (0.7199, 0.9286)	0.8654 (0.7826, 0.9273)
Logistic Regression	0.0654	0.9265 (0.8753, 0.9777)	0.8824 (0.8233, 0.9328)	0.7612 (0.6264, 0.8655)	0.8333 (0.7199, 0.9286)	0.9231 (0.8485, 0.9844)	0.9000 (0.8043, 0.9778)	0.8696 (0.7794, 0.9467)	0.9000 (0.8043, 0.9778)	0.8333 (0.7199, 0.9286)	0.8654 (0.7826, 0.9273)
Deep Neural Network	0.0499	0.9561 (0.9237, 0.9886)	0.8739 (0.8067, 0.9246)	0.7453 (0.6117, 0.8493)	0.8519 (0.7499, 0.9375)	0.8923 (0.8125, 0.9611)	0.8679 (0.7727, 0.9493)	0.8788 (0.7869, 0.9500)	0.8679 (0.7727, 0.9493)	0.8519 (0.7499, 0.9375)	0.8598 (0.7778, 0.9244)
C5.0 Decision Tree	0.0192	0.9510 (0.9153, 0.9867)	0.8739 (0.8067, 0.9244)	0.7453 (0.6124, 0.8487)	0.8519 (0.7546, 0.9394)	0.8923 (0.8055, 0.9571)	0.8679 (0.7692, 0.9464)	0.8788 (0.7910, 0.9516)	0.8679 (0.7692, 0.9464)	0.8519 (0.7546, 0.9394)	0.8598 (0.7810, 0.9216)
Naive Bayes	0.035	0.9450 (0.9069, 0.9832)	0.8739 (0.8067, 0.9244)	0.7429 (0.6114, 0.8484)	0.7963 (0.6773, 0.8983)	0.9385 (0.8750, 0.9874)	0.9149 (0.8250, 0.9821)	0.8472 (0.7571, 0.9242)	0.9149 (0.8250, 0.9821)	0.7963 (0.6773, 0.8983)	0.8515 (0.7640, 0.9177)
Ridge Regression	0.0686	0.9348 (0.8900, 0.9796)	0.8739 (0.8067, 0.9244)	0.7437 (0.6032, 0.8484)	0.8148 (0.6923, 0.9107)	0.9231 (0.8493, 0.9841)	0.8980 (0.8000, 0.9783)	0.8571 (0.7606, 0.9355)	0.8980 (0.8000, 0.9783)	0.8148 (0.6923, 0.9107)	0.8544 (0.7647, 0.9174)
Elastic Net	0.0658	0.9342 (0.8891, 0.9793)	0.8739 (0.8067, 0.9244)	0.7437 (0.6032, 0.8484)	0.8148 (0.6923, 0.9107)	0.9231 (0.8493, 0.9841)	0.8980 (0.8000, 0.9783)	0.8571 (0.7606, 0.9355)	0.8980 (0.8000, 0.9783)	0.8148 (0.6923, 0.9107)	0.8544 (0.7647, 0.9174)
Gaussian Process	0.0528	0.9564 (0.9256, 0.9873)	0.8655 (0.7983, 0.9244)	0.7271 (0.5944, 0.8467)	0.8148 (0.7069, 0.9138)	0.9077 (0.8305, 0.9718)	0.8800 (0.7777, 0.9649)	0.8551 (0.7639, 0.9345)	0.8800 (0.7777, 0.9649)	0.8148 (0.7069, 0.9138)	0.8462 (0.7640, 0.9143)
Flexible Discriminant Analysis	0.0333	0.9425 (0.9035, 0.9814)	0.8655 (0.8067, 0.9244)	0.7279 (0.5998, 0.8399)	0.8333 (0.7254, 0.9231)	0.8923 (0.8103, 0.9546)	0.8654 (0.7727, 0.9412)	0.8657 (0.7812, 0.9420)	0.8654 (0.7727, 0.9412)	0.8333 (0.7254, 0.9231)	0.8491 (0.7706, 0.9138)
Partial Least Squares DA	0.0756	0.9405 (0.8992, 0.9817)	0.8655 (0.7983, 0.9244)	0.7262 (0.5882, 0.8408)	0.7963 (0.6833, 0.8974)	0.9231 (0.8493, 0.9841)	0.8958 (0.7963, 0.9773)	0.8451 (0.7500, 0.9231)	0.8958 (0.7963, 0.9773)	0.7963 (0.6833, 0.8974)	0.8431 (0.7500, 0.9072)
Linear Discriminant Analysis	0.0728	0.9370 (0.8934, 0.9807)	0.8655 (0.7983, 0.9244)	0.7262 (0.5869, 0.8319)	0.7963 (0.6731, 0.8974)	0.9231 (0.8493, 0.9841)	0.8958 (0.7955, 0.9778)	0.8451 (0.7499, 0.9286)	0.8958 (0.7955, 0.9778)	0.7963 (0.6731, 0.8974)	0.8431 (0.7527, 0.9060)
LASSO Regression	0.0637	0.9219 (0.8691, 0.9748)	0.8655 (0.7983, 0.9244)	0.7271 (0.5922, 0.8370)	0.8148 (0.6938, 0.9078)	0.9077 (0.8281, 0.9706)	0.8800 (0.7719, 0.9608)	0.8551 (0.7606, 0.9342)	0.8800 (0.7719, 0.9608)	0.8148 (0.6938, 0.9078)	0.8462 (0.7555, 0.9091)
Neural Network	0.0484	0.9239 (0.8791, 0.9688)	0.8571 (0.7897, 0.9160)	0.7141 (0.5704, 0.8285)	0.8889 (0.7906, 0.9623)	0.8308 (0.7313, 0.9118)	0.8136 (0.7077, 0.9032)	0.9000 (0.8103, 0.9683)	0.8136 (0.7077, 0.9032)	0.8889 (0.7906, 0.9623)	0.8496 (0.7692, 0.9091)
Quadratic Discriminant Analysis	0.0589	0.8999 (0.8423, 0.9574)	0.8067 (0.7395, 0.8739)	0.6033 (0.4650, 0.7415)	0.6852 (0.5600, 0.8085)	0.9077 (0.8333, 0.9702)	0.8605 (0.7436, 0.9574)	0.7763 (0.6765, 0.8684)	0.8605 (0.7436, 0.9574)	0.6852 (0.5600, 0.8085)	0.7629 (0.6593, 0.8519)
Conditional Inference Tree	0.0803	0.8709 (0.8059, 0.9360)	0.8067 (0.7311, 0.8739)	0.6191 (0.4791, 0.7494)	0.9259 (0.8430, 0.9828)	0.7077 (0.5970, 0.8197)	0.7246 (0.6060, 0.8286)	0.9200 (0.8332, 0.9811)	0.7246 (0.6060, 0.8286)	0.9259 (0.8430, 0.9828)	0.8130 (0.7193, 0.8837)
K-Nearest Neighbors	0.0925	0.8177 (0.7409, 0.8944)	0.7647 (0.6891, 0.8403)	0.5268 (0.3766, 0.6660)	0.7593 (0.6458, 0.8645)	0.7692 (0.6665, 0.8616)	0.7321 (0.6154, 0.8334)	0.7937 (0.6889, 0.8889)	0.7321 (0.6154, 0.8334)	0.7593 (0.6458, 0.8645)	0.7455 (0.6535, 0.8264)
Classification and Regression Tree	0.1243	0.7709 (0.6923, 0.8496)	0.6807 (0.5966, 0.7647)	0.3829 (0.2369, 0.5252)	0.9074 (0.8163, 0.9787)	0.4923 (0.3729, 0.6067)	0.5976 (0.4935, 0.7037)	0.8649 (0.7500, 0.9655)	0.5976 (0.4935, 0.7037)	0.9074 (0.8163, 0.9787)	0.7206 (0.6281, 0.8000)
Sparse Linear Discriminant Analysis	0.091	0.9328 (0.8852, 0.9803)	0.5882 (0.5042, 0.6807)	0.1003 (0.0231, 0.1967)	0.0926 (0.0200, 0.1765)	1.0000 (1.0000, 1.0000)	1.0000 (1.0000, 1.0000)	0.5702 (0.4867, 0.6638)	1.0000 (1.0000, 1.0000)	0.0926 (0.0200, 0.1765)	0.1695 (0.0417, 0.3000)

### Clinical application of the model

**Fig. 7:**
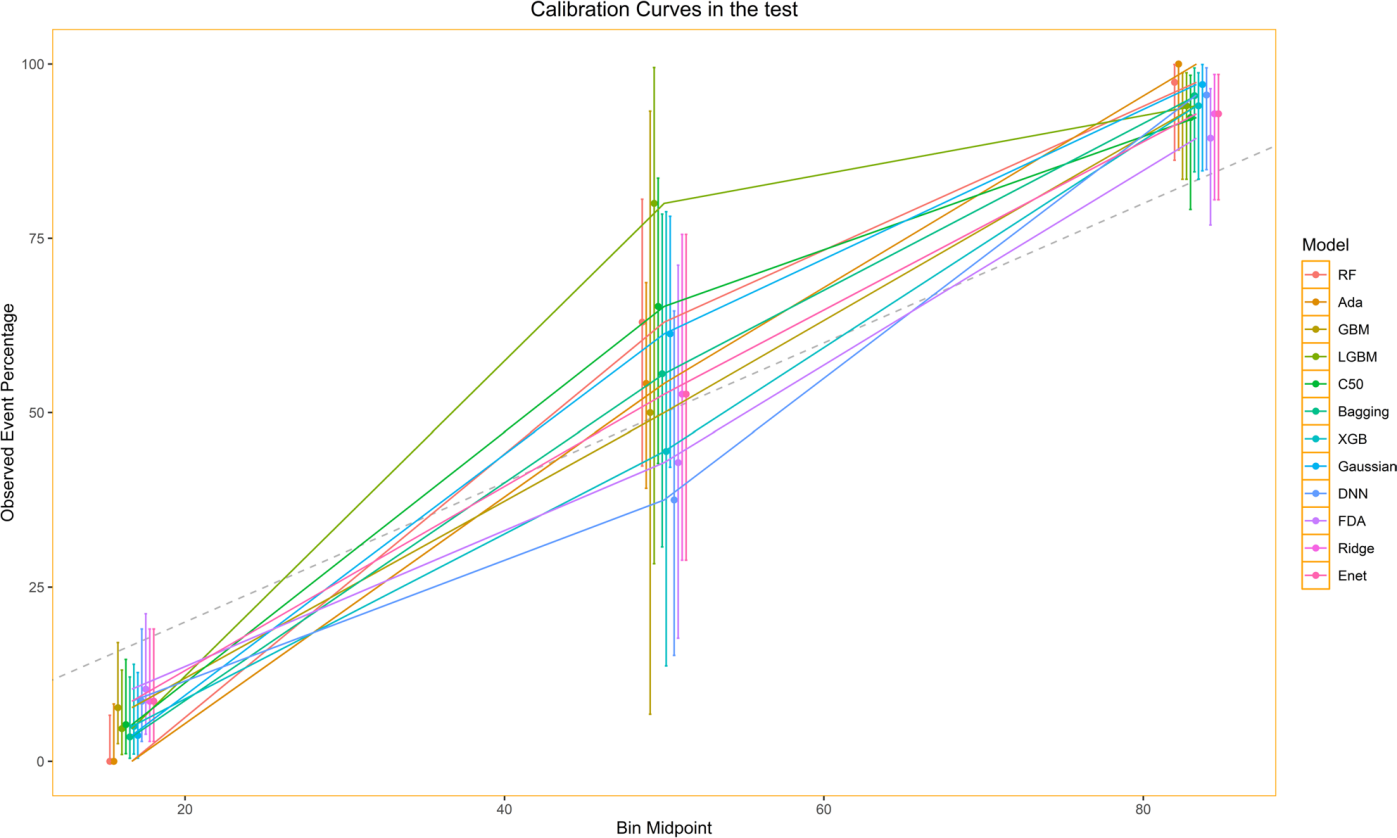
Calibration curves in the test

All models’ calibration curves were close to the ideal dashed line, and the curve-fitting quality of most models was high (see Fig. 7). The error bars (vertical lines) corresponding to each group were generally short, indicating smaller fluctuations in the actual event proportions within each group. This suggests that these predictive models had good calibration in the test set, with a high match between the model’s predicted death risk and patients’ actual 5-year mortality rates, further verifying the reliability of the model’s predictions.

To further quantitatively evaluate the calibration performance of the models, we employed two core metrics for the analysis: the Expected Calibration Error (ECE) and the Maximum Calibration Error (MCE). Specifically, ECE is the core metric for assessing global calibration performance, which reflects the weighted average deviation between the model-predicted probabilities and the actual event rates, with a smaller value indicating higher overall calibration accuracy. MCE serves as a supplementary metric for local calibration performance, which captures the maximum prediction deviation across all probability bins, where a smaller value corresponds to a lower risk of extreme model misprediction.

Quantitative results of calibration performance showed that the ECE of all models ranged from 0.0598 to 0.244. Among them, the XGB model achieved the lowest ECE (0.0598), representing the optimal global calibration accuracy; the ECE values of the LGBM, DNN, and GBM models were all below 0.08, demonstrating excellent overall calibration performance; the Ada model yielded the highest ECE (0.244), with relatively inadequate calibration performance. For MCE, the values of all models ranged from 0.1985 to 0.5541. The Gaussian model obtained the lowest MCE (0.1985), showing the best control effect on the risk of extreme misprediction, and the MCE values of the Ridge, Enet, LGBM and other models were also at a low level across all models. Overall, most of the included models presented favorable calibration capability, which can provide reliable support for the individualized prediction of 5-year mortality risk in patients.

**Fig. 8:**
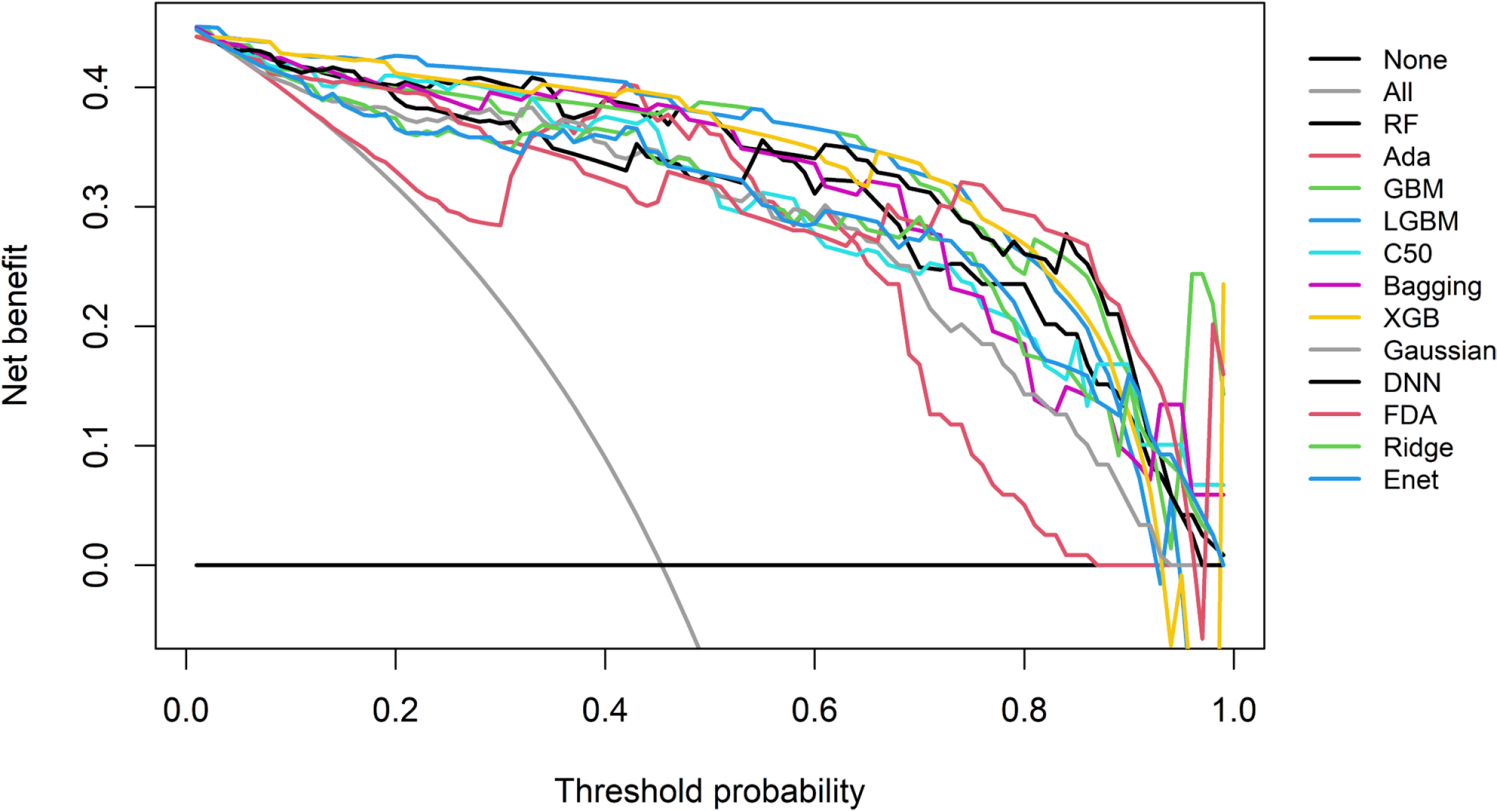
Decision curve analysis plot of 12 models

 Fig. 8 shows the results of clinical decision curve analysis for the 12 models. All models showed significantly higher net benefits than the two extreme strategies of “no intervention for any patient” (None) and “intervention for all patients” (All) within most clinical threshold probability ranges. Only a few models (e.g., FDA) showed slight decreases in net benefits at low thresholds. These machine learning models all provided substantial net benefits in the clinical decision scenarios of this study, and there were small differences in their clinical practical value, indicating that the practical value of the models in guiding clinical decision-making was superior to traditional experiential judgment.

### Model interpretability and clinical interactive application

We performed a global interpretability analysis of the prediction mechanism of the final LightGBM model using the SHAP method, with features ranked in descending order of global importance (defined as the mean of absolute SHAP values). The results showed that creatinine CRE, PSA, pT, and PSM were the top four core features with the highest contribution to the model’s predictions.

**Fig. 9:**
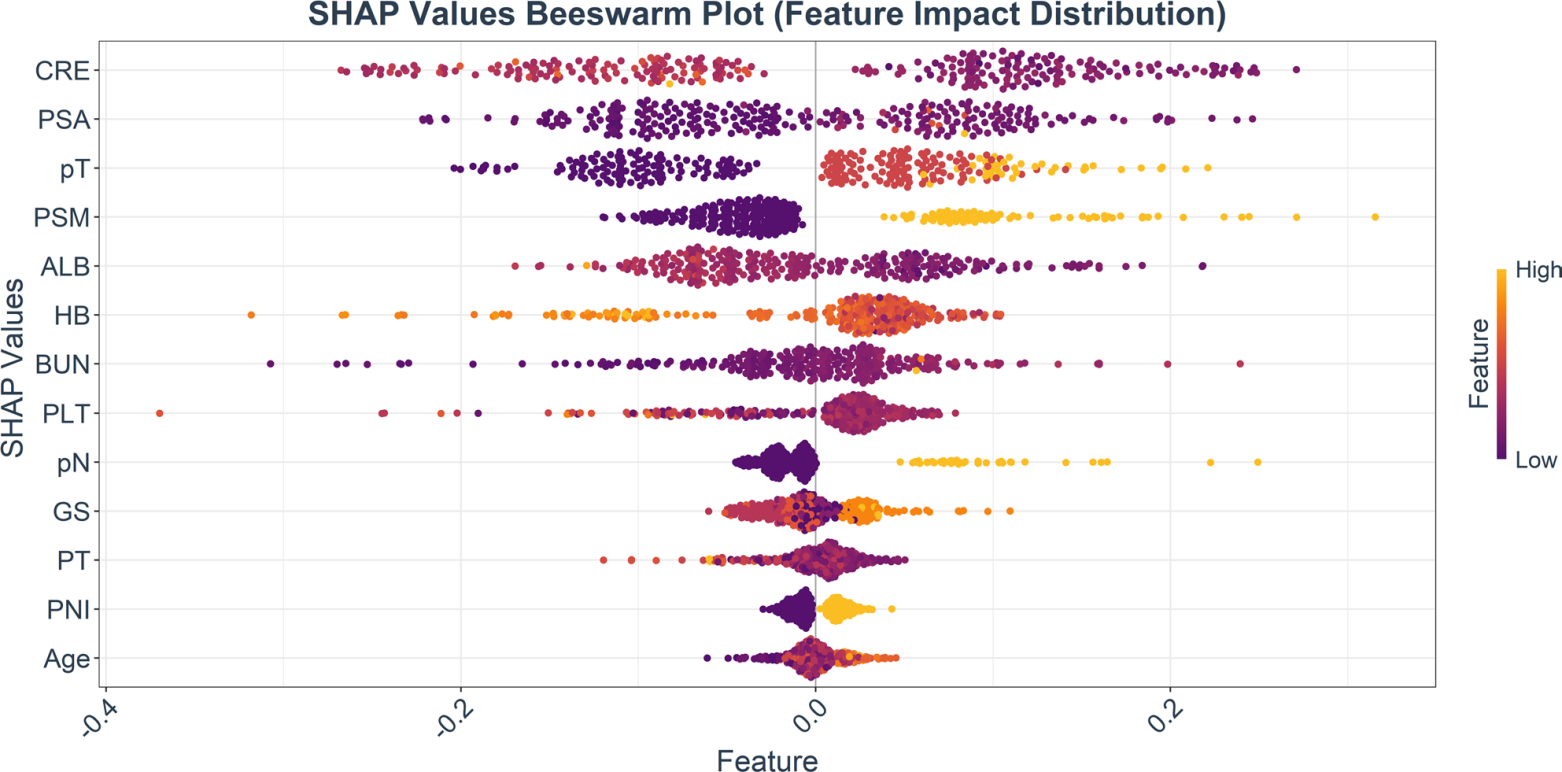
SHAP values of feature variables and their impact on prediction results

In terms of impact direction, CRE, pT, PSM, PSA, pN, GS, BUN, and Age were risk factors for mortality. Higher values of these features corresponded to more positive SHAP values, leading to an elevated 5-year mortality risk predicted by the model. In contrast, HB and ALB were protective factors against mortality: the higher the feature values, the more negative the corresponding SHAP values, and the lower the 5-year mortality risk predicted by the model. The SHAP values of PLT, PT, and PNI were concentrated around zero, exerting a weak overall impact on the model’s prediction results (see Fig. 9).

**Fig. 10:**
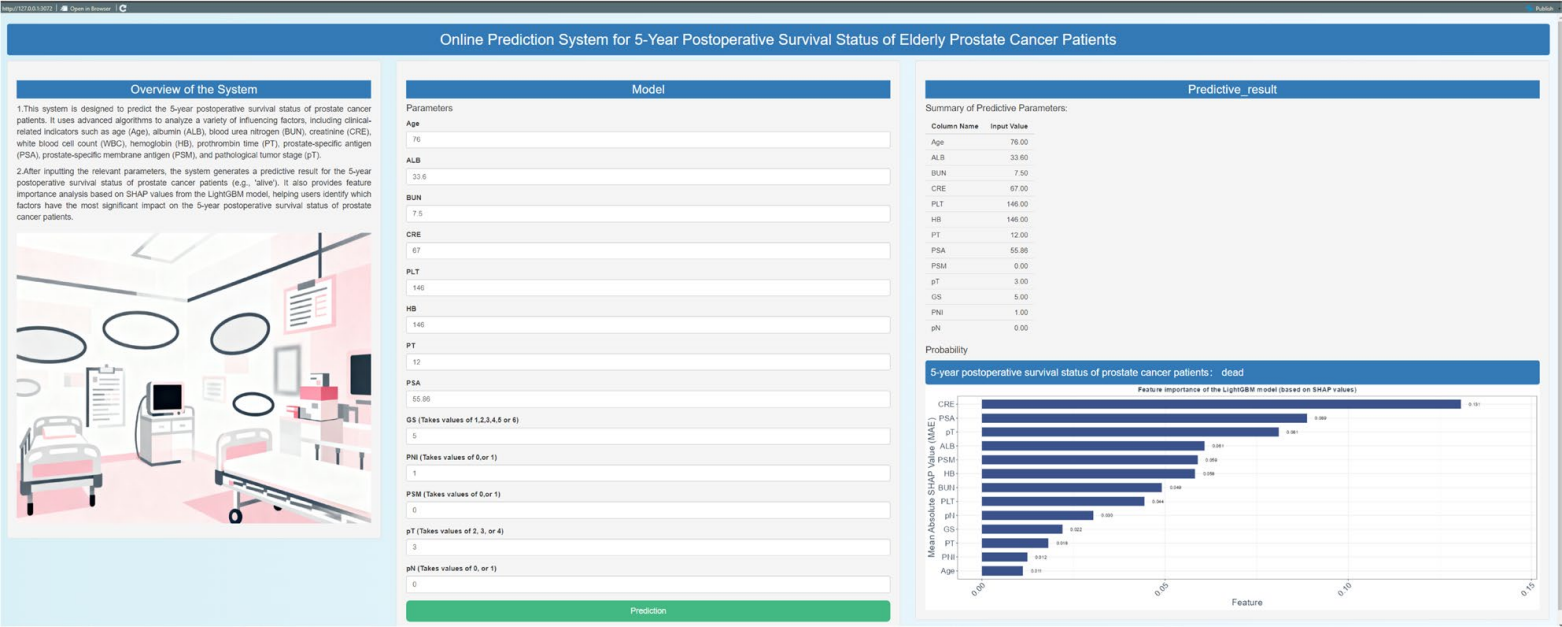
Online Prediction system for postoperative 5-year survival status of elder prostate cancer patients

This interactive web application developed with R Shiny (the tool shown in Fig. 10) is a predictive system designed specifically for prostate cancer patients to assess their 5-year postoperative survival status, equipped with multiple practical functions: it supports medical staff in entering 13 clinical and pathological parameters and conducts comprehensive analysis of these indicators through machine learning algorithms. In addition to outputting predictive conclusions on patients’ 5-year postoperative survival status, it also generates a visualization report of feature importance based on the SHAP values of the LightGBM model, helping medical staff intuitively identify which indicators (such as tumor stage, PSA level, etc.) have a more significant impact on survival prognosis.

The online prediction system features a concise, user-friendly interface. After completing parameter input, the medical staff only needs to click the “Predict” button to quickly obtain a comprehensive evaluation report including predictive results and feature analysis.

Currently, this application has been officially launched (access address: https://medicalpredictor.shinyapps.io/Online_Prediction_of_Risk_Factors/) and is free for medical staff to use. It can be directly integrated into the clinical evaluation workflow, providing data-driven support for clinical prognostic assessment.

## Discussion

Based on the data of prostate cancer patients from The First Affiliated Hospital of the University of Science and Technology of China (USTC), this study first systematically applied a variety of feature selection algorithms to screen 13 key predictive variables from a large number of potential predictors, and then constructed and validated a machine learning-based predictive model for the 5-year postoperative survival of prostate cancer patients. This streamlined and efficient survival assessment tool integrates multiple machine learning algorithms, enabling accurate, reliable, and personalized predictions.

In rigorous performance evaluation, the LightGBM model achieved excellent predictive performance for prostate cancer survival status, with an AUC of 0.9778, sensitivity of 0.9074, and specificity of 0.9538. This study conducted an in-depth analysis of the importance and influencing mechanisms of each variable using the SHAP algorithm, further clarifying the impact of different indicators on survival status.

The model demonstrates robust predictive performance in both validation and verification. It not only provides a feasible solution for individualized treatment evaluation of prostate cancer but also, through its post-operative 5-year survival prediction system, constructed using the LightGBM model, offers significant guidance for clinical practice. This system can provide data to help doctors formulate more precise, rational, individualized treatment plans for prostate cancer.

In recent years, some researchers have conducted AI-based prognostic studies on prostate cancer, though they mainly utilize clinical MRI and histopathological data.

Wong et al. analyzed 338 patients with localized prostate cancer who underwent robot-assisted prostatectomy. They employed three supervised machine learning algorithms and 19 distinct training variables (covering demographic, clinical, imaging, and surgical data) to construct a model in a hypothesis-free manner that predicts the risk of biochemical recurrence within 1 year after surgery [22]. Zhou et al. retrospectively collected data from 399 prostate cancer patients across three medical centers. They extracted the maximum regions of interest (ROI) from three MRI sequences, trained a deep learning model using H&E-stained sections, and built a nomogram for the combined model. By integrating multimodal data, they developed an AI-based predictive model for the progression of castration-resistant prostate cancer (CRPC) [23].

Lee et al. established a model integrating clinical data and deep learning to predict long-term biochemical recurrence-free survival (BCRFS) in PCa patients following radical prostatectomy (RP), utilizing multiparametric magnetic resonance imaging (mpMRI) [24]. Hou et al. initially developed MRI radiomics features (RadS) for biochemical recurrence (BCR), and employed two pre-specified AI models to forecast the tumor’s T3 stage and lymph node metastasis (LN+). Subsequently, clinical, imaging, and histopathological variables were incorporated into iBCR-Net for BCR prediction [25].

Polymeri et al. developed an AI-driven algorithm to automatically quantify the prostate and its tumor content in PET/CT images of 145 patients. This algorithm automatically acquired prostate tumor volume, tumor-to-prostate ratio, whole-tumor lesion uptake rate, and SUVmax values. Subsequently, the Cox proportional hazards regression model was applied to explore the correlations between these measurements and age, PSA, Gleason score, as well as prostate cancer-specific survival (PCSS) [26]. Koo et al. adopted an artificial neural network (ANN) model to predict survival outcomes under different initial treatment regimens and established an online decision-support system. Compared with the Cox proportional hazards regression model, the ANN model exhibited superior predictive performance in forecasting 5-year and 10-year progression-free survival (PFS) in castration-resistant prostate cancer (CRPC), cancer-specific survival (CSS), and overall survival (OS) [27].

The aforementioned literature has laid a solid foundation for prostate cancer research by integrating clinical, imaging, and pathological data to provide diverse approaches for model construction and clinical references for different prognostic objectives. Compared with previous studies, this study simplified data sources, developed a more user-friendly survival prediction model, and conducted a comprehensive and detailed comparison and optimization of various machine learning models. Through the study, it was found that T-stage, PSA, BUN, CRE, and Age have a significant impact on the 5-year survival status of prostate cancer patients after surgery. From Table 1, it was observed that at the T2 stage, the survival and death rates were 186 (62.8%) and 63 (21.3%), respectively, with the number of survivors significantly higher than that of non-survivors. However, at the T4 stage, the survival and death rates were 1 (0.3%) and 75 (25.3%), respectively, indicating an extremely poor prognosis.

PSA levels serve as a key basis for prostate cancer monitoring and are critical to its progression [28]. Fluctuations in PSA levels may act as an early indicator of BCR, rendering continuous PSA monitoring a vital step in tracking changes in prostate cancer status [29]. However, PSA level changes are not always directly associated with cancer recurrence. For instance, following certain specific treatments (e.g., high-intensity focused ultrasound, HIFU), PSA levels may surge sharply, which does not directly reflect the actual prostate cancer status [30, 31]. Thus, it is essential to acknowledge the limitations of PSA testing, as these can lead to unnecessary additional examinations and treatments, further intensifying patients’ psychological distress and increasing medical expenses [32].

In addition, we observed a discrepancy in the direction of the prognostic effect of CRE between the univariate logistic regression analysis and the SHAP interpretability analysis of the multivariable LightGBM model. In the univariate logistic regression, creatinine showed a protective effect against mortality (OR = 0.92, *P* < .001), which represents a crude unadjusted association. Specifically, creatinine levels are strongly correlated with patients’ nutritional status and muscle mass, and low creatinine levels are mostly accompanied by cancer-related malnutrition and cachexia, which explains the protective effect observed in the univariate analysis. In contrast, the SHAP analysis of the LightGBM model reflects the independent net effect of creatinine on the outcome after adjusting for confounding factors including nutritional status, tumor burden, and age. In this adjusted context, elevated creatinine independently indicates renal impairment, and renal insufficiency reduces patients’ tolerance to anti-tumor therapy and increases the risk of complications, making it an independent risk factor for poor prognosis in patients with prostate cancer.

A large-scale study of 160,787 U.S. men who underwent radical prostatectomy revealed that patients diagnosed at an older age had a higher prostate cancer mortality rate [33]. Among prostate cancer patients aged 75 years and above, cryotherapy demonstrated superior survival outcomes compared to other surgical methods for this specific age group [34]. Nevertheless, academic debate persists regarding the impact of age on prostate cancer mortality risk. A study by Pettersson et al. found no significant inherent effect of age on prostate cancer mortality risk; instead, it indicated that in current clinical practice, older prostate cancer patients may not receive adequate diagnostic evaluations and subsequent radical treatments [35].

Therefore, when formulating treatment plans for prostate cancer, it is necessary to develop individualized and precise plans based on the specific conditions of each patient. Particularly for the older prostate cancer population, given the extension of life expectancy, it is crucial to strictly adhere to the screening and treatment guidelines for prostate cancer and determine the most suitable individualized treatment strategies for them.

In this study, the LightGBM model exhibited superior performance to other machine learning algorithms. As a highly efficient gradient boosting framework, LightGBM not only excels in capturing complex nonlinear relationships and interactions among variables but also attains faster training speeds and reduced memory usage via histogram-based algorithms and one-sided gradient sampling techniques [36].

Machine learning methods offer significant advantages over traditional statistical methods for addressing complex, nonlinear clinical problems. The core innovation of our approach lies in the organic integration of model interpretability and clinical utility. Through SHAP value analysis, we can clearly identify the key factors influencing individual risk prediction, thereby enhancing clinicians’ trust in the model’s results and supporting the development of targeted intervention strategies [37]. This interpretability is crucial for the clinical implementation of machine learning models in decision support systems.

Furthermore, the interactive web app we developed converts complex machine learning outputs into intuitive clinical tools, enabling real-time risk evaluation and visualization of feature significance, thereby effectively addressing the “black box” issue of machine learning models [38]. Notably, this user-friendly interface design is consistent with Shah et al.‘s findings, highlighting its pivotal role in promoting the clinical implementation of machine learning technologies [39].

Machine learning methods offer significant advantages over traditional statistical methods for addressing complex, nonlinear clinical problems. The core innovation of our approach lies in the organic integration of model interpretability and clinical utility. Through SHAP value analysis, we can clearly identify the key factors influencing individual risk prediction, thereby enhancing clinicians’ trust in the model’s results and supporting the development of targeted intervention strategies [37]. This interpretability is crucial for the clinical implementation of machine learning models in decision support systems.

In addition, the interactive web application developed by our team converts the complex outputs of machine learning into an intuitive clinical tool, which enables real-time risk assessment and feature importance visualization. It effectively mitigates the black-box problem of machine learning models in clinical prediction, though there is still room for improvement regarding the comprehensiveness of interpretation [38]. Notably, this user-friendly interface design is consistent with Shah et al.‘s findings, highlighting its pivotal role in promoting the clinical implementation of machine learning technologies [39].

This study employs machine learning methods combined with the SHAP tool to construct a predictive model, thereby enhancing its interpretability. By providing real-time predictions and clinical recommendations, it helps clinicians make more accurate, personalized treatment decisions. However, this study has the following limitations: First, the dataset was based on retrospective clinical data, which may lack dynamic monitoring indicators, such as long-term follow-up data on patients’ quality of life, treatment compliance, and adverse reaction records. Meanwhile, incomplete inclusion of lost-to-follow-up patients has led to potential selection bias. Furthermore, the total sample size of this study was 300 cases, which is relatively limited, and all samples were collected from a single center. This may compromise the external validity and generalizability of the model, whose applicability to multi-center and multi-ethnic populations requires further verification. Moreover, the current model was constructed based on routine clinical indicators (e.g., T stage, prostate-specific antigen, blood urea nitrogen, creatinine, and age), without incorporating imaging data (e.g., magnetic resonance imaging and computed tomography) and genomic data (e.g., gene mutation status). Thus, the comprehensiveness and depth of the feature variables need to be further improved. Finally, this study focused on predicting the 5-year survival status, and the predictive efficacy of the model for long-term survival (e.g., 10-year survival) has not been verified. Future research should extend the follow-up duration and supplement follow-up data to optimize the model.

To address these aforementioned issues, the research team aims to expand and enrich the dataset by integrating and harmonizing multi-source data, encompassing imaging data, genomic profiles, and other granular clinical covariates to construct a more comprehensive and precise predictive model. In turn, this model will offer more robust evidentiary support for prostate cancer treatment decision-making, thereby enhancing the scientific rigor and clinical translatability of individualized treatment strategies and prognostic assessment [40].

## Conclusion

This study conducted relevant explorations on predicting the survival of prostate cancer patients using multiple machine learning models. Among these machine learning models, the LightGBM model exhibited the best comprehensive performance with an AUC value of 0.9778, indicating that this model has good efficacy in predicting treatment regimens for prostate cancer within the scope of the dataset used in this study. This study analyzed the internal mechanism of the LightGBM model using the SHAP tool and found that five indicators, namely T stage, CRE, PSA, PSM, and ALB, were significantly correlated with the 5-year postoperative survival rate of prostate cancer patients. By integrating machine learning algorithms with SHAP interpretability analysis, this study constructed a model applicable for predicting treatment regimens for prostate cancer. Its predictive efficacy has been verified to reach a high level in this study, providing potential reference and technical support for the clinical implementation of precise individualized decision-making.

## Supplementary Information

Supplementary Material 1.

Supplementary Material 2.

Supplementary Material 3.

Supplementary Material 4.

## Data Availability

Data are available from the corresponding author upon reasonable request.
