# Supplementary figures and images for: Explainable and visualizable machine learning model development and validation for 5-year postoperative survival prediction in prostate cancer patients aged ≥ 65 years
Source: BMC Geriatr. 2026 May 4;26:725. doi: 10.1186/s12877-026-07551-2 (PMC13195998; doi:10.1186/s12877-026-07551-2)

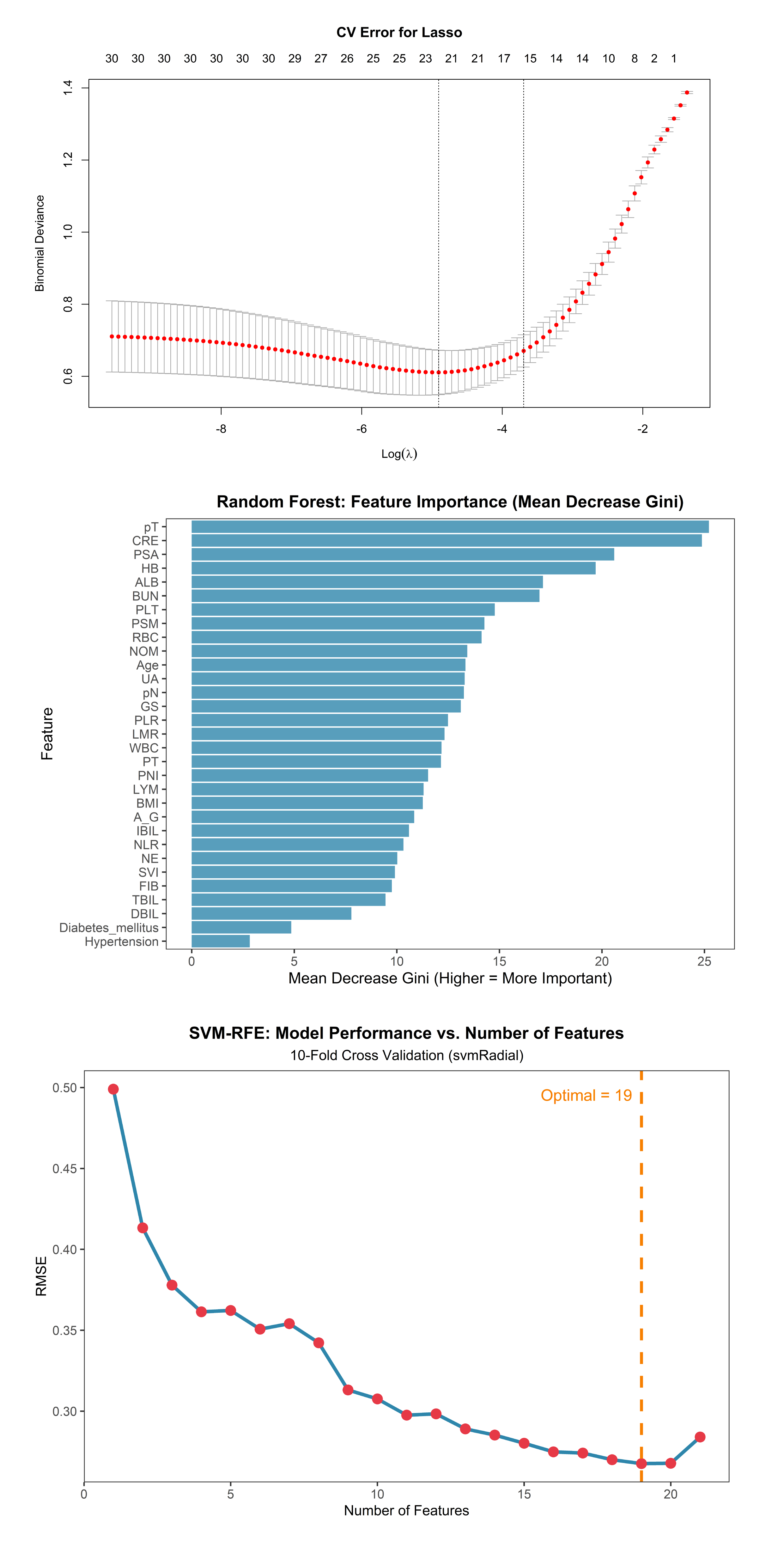

Supplement: Supplementary file 2 — Supplementary Material 2. [file 12877_2026_7551_MOESM2_ESM.tif]

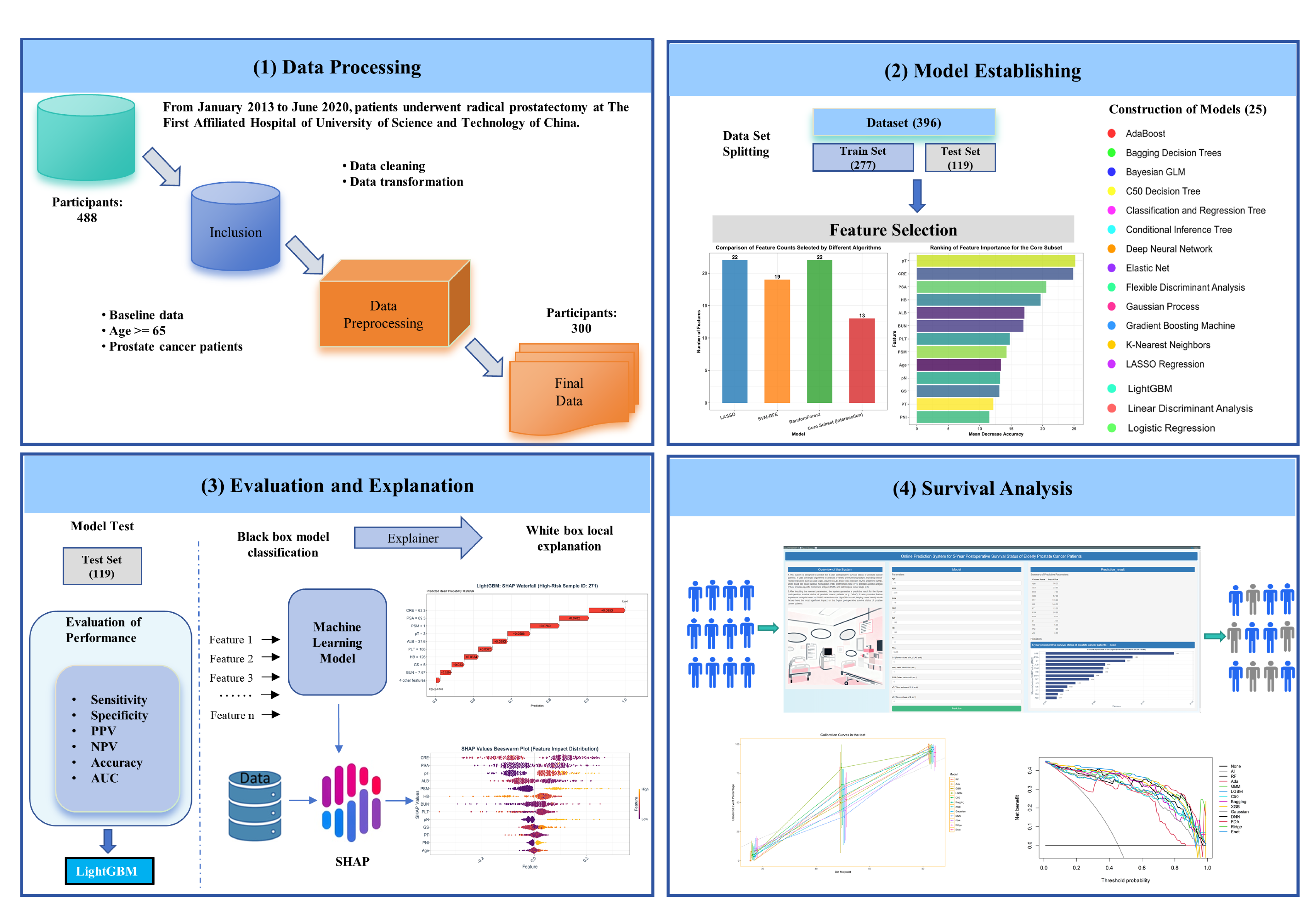

Supplement: Supplementary file 4 — Supplementary Material 4. [file 12877_2026_7551_MOESM4_ESM.tif]
